# From chip to SNP: Rapid development and evaluation of a targeted capture genotyping-by-sequencing approach to support research and management of a plaguing rodent

**DOI:** 10.1371/journal.pone.0288701

**Published:** 2023-08-17

**Authors:** Kevin P. Oh, Nikki Van de Weyer, Wendy A. Ruscoe, Steve Henry, Peter R. Brown

**Affiliations:** 1 Applied BioSciences, Macquarie University, Sydney, NSW, Australia; 2 CSIRO Health & Biosecurity, Canberra, ACT, Australia; University of Veterinary Medicine Vienna: Veterinarmedizinische Universitat Wien, AUSTRIA

## Abstract

The management of invasive species has been greatly enhanced by population genetic analyses of multilocus single-nucleotide polymorphism (SNP) datasets that provide critical information regarding pest population structure, invasion pathways, and reproductive biology. For many applications there is a need for protocols that offer rapid, robust and efficient genotyping on the order of hundreds to thousands of SNPs, that can be tailored to specific study populations and that are scalable for long-term monitoring schemes. Despite its status as a model laboratory species, there are few existing resources for studying wild populations of house mice (*Mus musculus* spp.) that strike this balance between data density and laboratory efficiency. Here we evaluate the utility of a custom targeted capture genotyping-by-sequencing approach to support research on plaguing house mouse populations in Australia. This approach utilizes 3,651 hybridization capture probes targeting genome-wide SNPs identified from a sample of mice collected in grain-producing regions of southeastern Australia genotyped using a commercially available microarray platform. To assess performance of the custom panel, we genotyped wild caught mice (N = 320) from two adjoining farms and demonstrate the ability to correctly assign individuals to source populations with high confidence (mean >95%), as well as robust kinship inference within sites. We discuss these results in the context of proposed applications for future genetic monitoring of house mice in Australia.

## Introduction

Invasive alien pest species pose significant threats due to persistent and widespread impacts on endemic biodiversity, human health, and economic enterprise. The management of invasive species has been greatly enhanced over the last several decades by the application of population genetic analyses, which can provide valuable information regarding pest population structure, invasion pathways, and reproductive biology [1]. More recently, advances in molecular technologies have made possible the analysis of genome-wide variation, most commonly by genotyping large numbers (e.g., typically thousands to millions of loci in vertebrate genomes) of single-nucleotide polymorphisms (SNPs) across invasive populations at regional and continental scales [2]. The resulting SNP datasets have been used to improve resolution and the ability to detect fine-scale population genetic structure and identify sources of new outbreaks [1, 3], infer landscape effects on gene flow [4], screen for genetic resistance to chemical-based control agents [5, 6], and inform the development of future pest control technologies [7, 8].

Despite these potential benefits, the application of genomic approaches to problems in invasive species management is often hindered by constraints of available SNP genotyping techniques. While whole-genome (re)sequencing provides the most comprehensive view of genetic variation and has become increasingly accessible in recent years, it is still often prohibitively expensive for mammalian or larger-sized genomes and for long-term monitoring programs that involve widespread and repeated sampling over time, and resulting datasets place large demands on computation and data storage infrastructure. In comparison, reduced-representation sequencing approaches (e.g., RAD-seq [9]) offer greater efficiencies by subsampling of the genome, typically through the use of restriction enzyme digestion [10]. However, such methods may often involve complex laboratory protocols, are constrained to sequencing markers that occur near restriction sites, and can suffer from lack of consistency between samples and studies due to variation in sample DNA degradation, sequencing batch artefacts, and null alleles due to polymorphisms at cut sites [11, 12], thereby reducing the final number of useable markers in the final dataset. Still another option is commercial array-based genotyping platforms, which offer rapid and highly robust genotyping at 10,000s to 100,000s of SNPs with minimal lab preparation, and which has seen some recent applications in invasive wildlife research contexts [13]. However, due to the high costs of development, these arrays are typically restricted to laboratory model organisms or domesticated species, or closely related taxa [14]. Moreover, the design of such arrays is largely based on variation segregating in laboratory lines or livestock breeds, which may not be relevant or can even lead to skewed population genetic inferences when applied to wild populations [15]. Thus, while many of these approaches may be suitable for some systems, there is still commonly a need for protocols that offer efficient and reliable genotyping on the order of hundreds to thousands of SNPs, that can be tailored to specific study populations and that can be practically scaled to accommodate a range of sampling schemes [16].

Methods that allow effort to be targeted on specific genomic regions of interest (e.g., regions that harbor genes of interest or informative SNPs) can greatly increase sequencing and bioinformatic analysis efficiency compared to whole-genome resequencing [17]. Hybridization capture sequencing refers to a genotyping-by-sequencing method wherein a panel of custom oligonucleotide probes, designed to hybridize to specific complementary sequences in the genome, are used to enrich genomic libraries prior to sequencing. In this regard, hybridization capture is similar to other targeted methods like amplicon sequencing, though technical limitations affect suitability of these methods for different applications, with hybridization capture typically recommended for studies involving thousands of markers across hundreds of samples, whereas amplicon sequencing is better suited for genotyping hundreds of markers across thousands of samples [18]. One key advantage of in-solution hybridization sequencing approaches is the ability to easily modify panels through the addition or removal of individual probes with little impact on overall assay performance, thereby allowing on-going customizations that can sometimes cause problems with highly multiplexed amplicon-based approaches. Compared to most other reduced-representation sequencing techniques, these targeted sequencing methods allow for precise tuning of the genotyping panel and greater marker reproducibility among labs and experiments. Consequently, these techniques have attracted attention for population genetics applications in conservation and wildlife management [18, 19], though there are comparatively fewer examples in applied management of invasive pests.

Here we present the development and evaluation of a targeted capture SNP genotyping approach for population genetic analyses of invasive house mice (*Mus musculus* spp.) in southeastern Australia that are notable for aperiodic eruptions in population numbers that can lead to ‘mouse plagues’ [20]. These outbreaks disrupt ecosystems, cause significant damage to agriculture and other economic enterprise, and pose health threats to domesticated animals and humans [21, 22]. Despite its status as a model system for understanding outbreak ecology, there still exist considerable gaps in understanding population structure and connectivity, the ecology of house mice in broadacre cropping habitat [23], and the role of kin structure in regulating population dynamics [24]. For many of these research questions, population genetic analyses of high-density SNP datasets are likely to contribute important new insights. Moreover, there are often direct management applications for SNP genotyping such as identifying the sources of new mouse outbreaks at fine geographic scales, and understanding the prevalence of alleles conferring resistance to commonly utilized anticoagulant rodenticides [25]. Ideally, there would be a single versatile SNP genotyping panel that could be applied across any number of these research questions.

Despite its status as a model laboratory species, there are surprisingly few established high-density SNP genotyping options well-suited for research of wild house mouse populations. The third generation of the Mouse Universal Genotyping Array, GigaMUGA [26], is a microarray-based genotyping platform that consists of probes targeting 141,090 high-quality single-nucleotide polymorphisms (SNPs), the majority of which were designed to be informative across laboratory lines, with a smaller fraction (20,237) selected based on expected polymorphism within wild mouse populations. The chip array also includes probes targeting particular genes of interest including two SNPs in the *VKORC1* gene that are associated with resistance to the warfarin, an anticoagulant rodenticide registered for use in Australia and therefore expected to strongly favor such mutations, as has been observed in other countries [27]. Veale et al. [28] were the first to use GigaMUGA on wild house mice for their study of genomic admixture and population structure of mice in New Zealand along with some samples from Australia. Their results indicated a high proportion of successful SNPs genotyped (>90%), though less than half of these remained after pruning of markers that were in linkage disequilibrium. More recently, Morgan et al. [29] found similar results when applying the genotyping array to a population genetic study of house mouse populations spanning five continents. However, the potential utility of GigaMUGA for supporting research and management of plaguing house mice populations at smaller spatial scales has yet to be evaluated.

In the present study we perform population genetic analyses using GigaMUGA genotypes from a sample of mice collected from grain-growing regions of southeastern Australia. We then use these data to inform design of a custom hybridization capture panel, which is utilized to genotype a larger sample set of mice collected from two adjacent farms. We evaluate the ability of this approach for addressing key questions in pest research and management applications, including identification of source populations, screening for rodenticide resistance alleles, and inference of kin structure. We discuss the result with respect to applicability for ongoing genetic monitoring to inform management across these agricultural regions.

## Materials and methods

### Animal ethics statement

The use of animals in this study was carried out in strict accordance with the Australian code for the care and use of animals for scientific purposes (8^th^ ed., 2013) and all protocols were reviewed and approved by the CSIRO Wildlife, Livestock and Laboratory Animal, Animal Ethics Committee (approvals 2018–33, 2019–18, and 2020–24). All mice were live-trapped, and released at the point of capture as soon as possible following visual confirmation of no adverse effects.

### Sample collection and preparation

**Fig 1:**
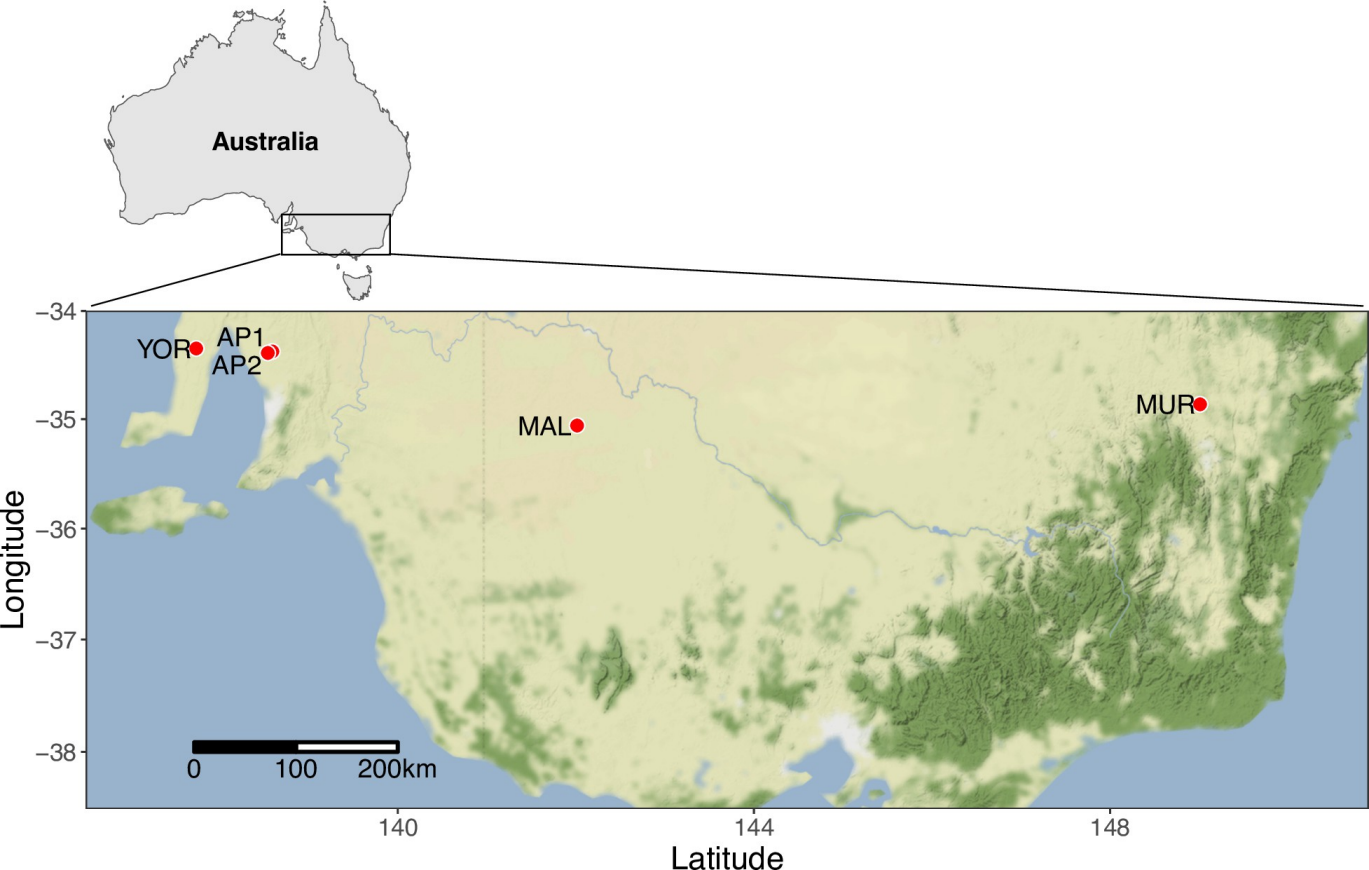
Sampling locations of house mice trapped for SNP genotyping (2019–2020). See methods for location abbreviations.

Tissue samples were obtained from live-trapped mice using Longworth box traps (Longworth Scientific, Abingdon, UK) at two agricultural study sites in the Adelaide Plains of South Australia (AP1 and AP2, N = 320) along with an additional 24 individuals from other sites in southeastern Australia: Northern Mallee, Victoria (MAL, N = 7); the Yorke Peninsula, South Australia (YOR, N = 8), and near Murrumbateman, New South Wales (MUR, N = 8) (Fig 1). These sites were all located near or within active commercial farms (primarily growing wheat and barley). Traps were supplied with polyester fiber bedding, baited with wheat grain, and set in the afternoon (mice on these sites are typically active aboveground only from dusk until dawn). Traps were checked before sunrise the following morning and any captured animals were immediately removed by trained technicians. Ear snips (2-3mm of the distal pinna) were excised using sterilized surgical scissors and stored in 70% ethanol for later processing. Mice were individually marked with RFID passive integrated transponder tags (Biomark MiniHPT10, Merck and Co., Inc.) using a 16 gauge needle prior to release.

Genomic DNA was isolated using column-based methods (DNeasy Blood and Tissue Kits, Qiagen, Inc.) following the manufacturer’s recommended protocol. DNA concentration was quantified with a Qubit 3 fluorometer (Thermo Fisher Scientific Inc.) and purity was assessed by inspecting the A260/A280 ratio for each sample on a NanoDrop ND-1000 spectrophotometer (Thermo Fisher Scientific Inc.).

### GigaMUGA array genotyping and analysis

In order to assess genetic variation across all study sites, we selected a subset of purified DNA samples (N = 72) that included 48 samples randomly selected from the two South Australia sites along with all 24 samples from the other sites. These samples were submitted to Neogen, Inc. (Lincoln, NE, USA) for genotyping on the GigaMUGA [26] Illumina Infinium II array (Illumina Inc.). Genotypes were initially called using Illumina BeadStudio/GenomeStudio software. The argyle R package [30] was subsequently used for genotype quality control following threshold quantile normalization of raw intensity values (function tQN). Genotypes were then filtered for minimum minor allele frequency (MAF > 0.05) and subjected to linkage disequilibrium pruning (window size 1kb, step size 1, R^2^ = 0.5) in PLINK v1.9 [31]. To evaluate population genetic structure, principal components analysis (PCA) was carried out using the adegenet R package [32] and visualized on a biplot.

### Custom targeted capture panel design

To select a reduced set of markers from the GigaMUGA dataset that were informative for variation among our study sites, we performed discriminant analysis of principal components (DAPC, [33]) on the filtered set of genotypes (70 samples at 49,058 markers, see Results). Populations were defined a priori based on sampling location, and a cross-validation procedure (xval.dapc function) was carried out (1000 iterations) following recommended procedures to determine the optimal number of principal components to retain. To identify SNPs that contributed most to differentiation among mice at different sampling locations, we examined the loading of each locus to the first two discriminant linear functions identified by the DAPC analysis and selected markers in the 95^th^ percentile. This list of target SNPs was submitted to the oligonucleotide provider (Integrated DNA Technologies, Ltd.) for design of complementary probes (based on the mm10 reference genome) and final screening for any predicted off-target activity. 5’-biotinylated oligonucleotide probes (120 nucleotides length each) for the final panel design (3,651 SNPs, see Results) were synthesized and subsequently mixed in equimolar concentrations.

### Library preparation and sequencing

To evaluate the performance of our custom panel, we genotyped 320 individuals (including the same DNA extracted from 47 individuals that were genotyped in the GigaMUGA dataset) following the manufacturer’s recommended guidelines (xGen Hybridization Capture of DNA Libraries Protocol, IDT, Ltd.). Briefly, genomic libraries were prepared from 100 – 120ng of genomic DNA per sample using Lotus DNA Library Prep Kits (IDT, Ltd.) at 0.4x the standard reaction volume for cost savings. Each sample was subject to a single enzymatic preparation step that included enzymatic fragmentation at 32°C (to target a 200 bp average insert size), followed by end-repair and A-tailing at 65°C. Illumina-compatible stubby adapters were subsequently ligated to each sample, followed by PCR library amplification (9 cycles) with unique dual-indexed primers (IDT, Ltd.), to minimize effects of index-hopping. Libraries were quantified in triplicate by qPCR (NEBNext Library Quant Kit, New England Biolabs, MA, USA) then combined in equimolar concentrations into pools of 16 samples each in preparation for target capture. Pooled libraries were hybridized to probes in the presence of universal blocking oligos (xGen, IDT Ltd.) and mouse Cot-1 DNA (ThermoFisher, Inc.) to minimize off-target binding, and fragment capture was performed with washed streptavidin beads. Post-capture library amplification was carried out using high-fidelity PCR (KAPA HiFi HotStart DNA Polymerase, Roche) with xGen Library Amplification primers (IDT Ltd.) for 10 cycles, followed by clean-up using AMPure XP bead reagent (Beckman Coulter, Inc.) at 1.5X reaction volume. Mean fragment length of captured pooled libraries was assessed via automated electrophoresis using an Agilent 2200 TapeStation system (Agilent Technologies, Inc.) with high sensitivity D1000 reagents, and library quantification performed using qPCR (NEBNext Library Quant Kit, New England Biolabs, MA, USA) in triplicate at two concentrations for each library after serial dilution. These pools were then pooled together in equimolar concentrations to generate either 48-plex (i.e., combining three 16-plex captured library pools) or 96-plex (i.e., combining six 16-plex captured library pools) final sequencing libraries, which were sequenced on a MiSeq instrument (Illumina, Inc.) for 300 cycles (2 x 150 bp paired end) using v2 Micro and v2 standard flowcell kits, respectively, along with a 5% spike-in of PhiX control library.

### Bioinformatic processing and genotype evaluation

Quality checks of raw sequence data were carried out using FastQC (http://www.bioinformatics.babraham.ac.uk/projects/fastqc), followed by trimming and filtering of adapter sequences using BBTools (http://bbtools.jgi.doe.gov). Sequences were mapped to the mm10 reference genome using BWA-MEM2 [34] and SNP calling performed using the multiallelic model in BCFtools v1.12 [35]. SNPs were characterized and subsequently filtered for genotype quality and read depth using VCFtools 0.1.16 [36] and BEDtools v2.30.0 [37].

To evaluate concordance among sequencing and GigaMUGA datasets, we compared VCF files that include only shared sites (after basic quality filtering, but prior to any filtering for minor allele frequencies) using the concordance command in SnpSift v5.1d [38].

### Population genetic analysis and kinship inference

To characterize genetic variation and assess the ability to discriminate fine scale population genetic structure with the hybridization sequencing dataset, we performed PCA and DAPC on the Adelaide Plains (AP1 and AP2) samples (N = 320) using adegenet in R. These study sites are located in close proximity (~5km) on adjoining farms within homogenous cropping habitat with no obvious barriers to mouse movement. We reasoned that these sites would thus provide a challenging scenario to test the ability of the custom genetic marker panel to detect fine scale genetic differentiation and assign samples to source populations under conditions with probable gene flow, and at a scale that is likely relevant for farm-level management decisions. As the goal was to visualize genetic differentiation (as opposed to inferring genetic clusters de novo), DAPC was carried out with samples assigned a priori to populations, which has been shown to yield robust estimates of genetic distance [39]. Additionally, the cross-validation procedure provided in adegenet was performed as above, but in this instance the purpose was not only to identify the optimal number of principal components retained, but also to assess the ability to correctly identify source populations of individual mice. In this procedure, DAPC is performed on a randomly selected training subset of samples (90% from each population), which is then used to predict the source of the remaining 10% of samples. The mean rate of successful assignment is calculated, and the process is repeated iteratively (1000), thereby providing an estimate of dataset performance (given an optimal number of principal components retained) for this application.

Pairwise kinship coefficients among all mice in these study populations were estimated using PC-Relate [40] as implemented in the GENESIS R package [41], which estimates kinship coefficients whilst accounting for underlying population genetic structure. Resulting values were compared with estimates generated using the full GigaMUGA genotypes for the 47 samples that occurred in both datasets.

## Results

### GigaMUGA genotyping

**Fig 2:**
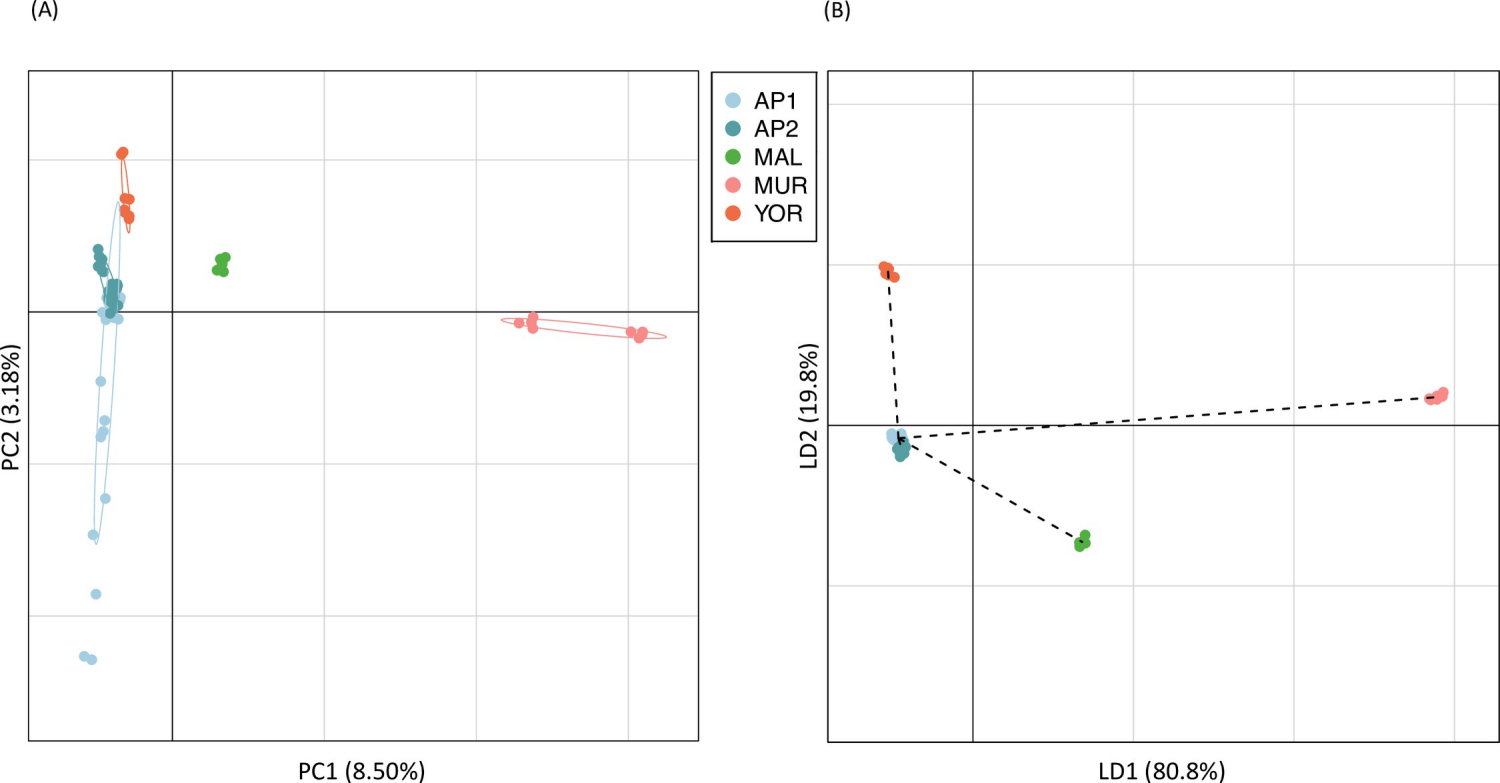
Analysis of population genetic variation based on filtered GigaMUGA genotypes (49,058 SNPs) from 70 mice sampled at five locations in southeastern Australia. (A) Results of PCA, with axes representing the first two principal components along with percentage of total genetic variation explained. Points are individual samples with inertia ellipses shown for each population. (B) Results of DAPC, which identifies synthetic variables that maximize the differences among populations. LD1 and LD2 are the first two linear discriminants, along with respective percentage of interpopulation variation explained. Population means are connected by dashed lines representing the minimum spanning tree based on the squared distances among populations.

Quality assessment of GigaMUGA genotypes resulted in the exclusion of two samples (≥10% missing SNP genotypes) and 8,918 markers (genotypes missing for ≥10% of samples). For initial assessment of genetic analyses that focused on autosomal loci, markers were further filtered for a minimum minor allele frequency of 0.05 (removed 54,425 loci, including 40,331 invariant markers) and linkage disequilibrium pruning (removed 25,562 markers), resulting in a final dataset of 49,058 markers (i.e., 35.7% of the original 137,475 autosomal sites in the array). PCA of these genotypes (Fig 2A) identified two leading axes (PC1, PC2) that explained 8.5% and 3.1% of genetic variation in the dataset, respectively, and largely reflected patterns of isolation by distance, with South Australia mice (AP1, AP2, and YOR) in near overlapping clusters, differentiated from mice from in central Victoria (MAL) and the even more distant MUR mice.

Of the 70 individuals retained in the analysis, all were homozygous for the reference allele at both SNPs in *VKORC1* (chr7:127,895,440 C>T and chr7:127,894,606 C>A) except a single individual that had a missing genotype at one locus.

### Design of hybridization capture panel

Results of the DAPC for the GigaMUGA dataset are summarized in Fig 2B (for further analysis details see S1 Fig). Based on output from the cross-validation procedure, we retained 34 principal components (accounting for 64.1% of genetic variation in the dataset) which maximized the average proportion of successful population assignments for individual samples (0.959) whilst also achieving the lowest root mean squared error (0.063). Four discriminant functions were retained in the final analysis, and the resulting leading axes (LD1 and LD2, Fig 2A) explained 80.8% and 10.8% of interpopulation variance in the data and largely mirrored patterns observed in the PCA.

Inspection of individual marker loadings on the discriminant functions identified a panel of 3,783 targeted SNPs that were submitted for 1x tiling oligonucleotide probe design. Quality assessment of this initial panel identified 132 sequences that were predicted to have moderate to high off-target activity and were thus excluded. The final pool of 3,651 markers included targets distributed across all 19 autosomes, with an average of 176.4 (S.D. = 45.3) markers per chromosome, along with 282 and 17 markers on the X and Y chromosomes, respectively (S1 Table). Probes for mitochondrial SNPs were excluded as targeting such loci would be expected to lead to overwhelming representation in enriched libraries.

### Hybridization capture sequencing

**Fig 3:**
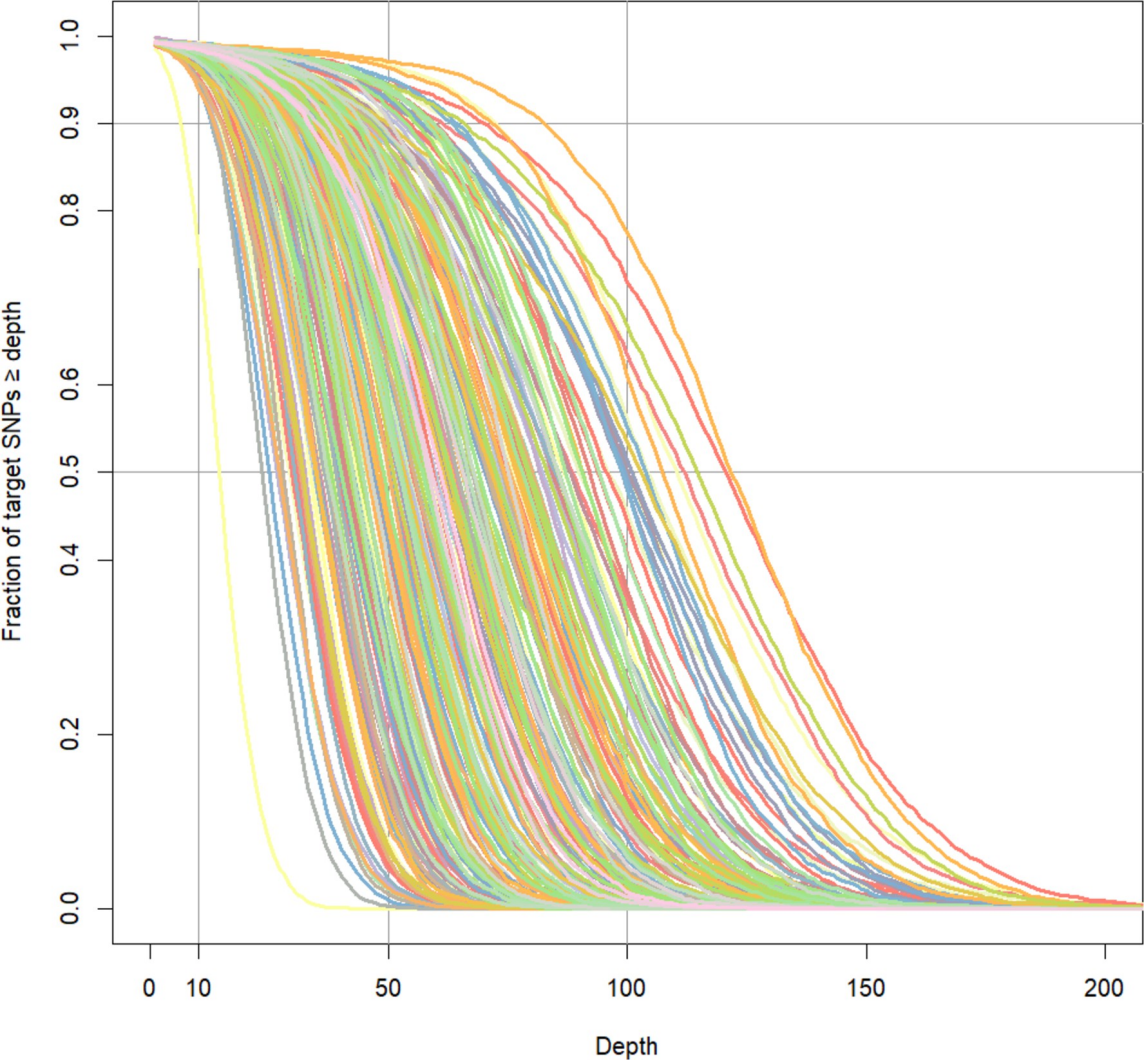
Target SNP sequencing coverage from hybridization sequence capture. Each curve represents a different sample (N = 317), and the observed proportion of targeted SNPs (3,651 total) that had coverage equal to or greater than the value indicated on the x-axis.

Analysis of mapped sequencing reads indicated successful capture of the target loci (Fig 3), with depth of coverage greater than 10x for >90% of targeted SNPs in the panel. Quality assessment of the called genotypes resulted in the exclusion of three samples that had ≥10% missing genotypes, whilst filtering of markers (genotype quality ≥30, missing genotypes across samples ≤10%, and minimum average read depth ≥10x) resulted in the exclusion of 86 loci, yielding overall dataset of 3,565 SNPs (i.e., 97.7% of the 3,651 targeted). Mean coverage at autosomal target SNPs across all samples was 27.6x (S.D. = 9.84).

### Comparison between genotyping methods

For the 47 samples that were genotyped with both the GigaMUGA and the hybridization capture sequencing panel, a total of 3,562 SNPs (97.6% of the targeted loci) were retained for comparison after initial quality filtering (see above). For these markers, we observed strong concordance between datasets, with 98.9% (165,544) of called genotypes identical, whereas only 0.66% (1,100 genotypes) were discordant (for details of discordant genotypes see S2 Table). While the overall genotyping success rate was high in both filtered datasets, we observed 8.15 times more missing genotypes in the GigaMUGA data at positions that were called in the hybridization capture sequencing dataset (375, or 0.22% of all genotypes) compared to the reciprocal direction (46, or 0.03% of all genotypes). We also observed 22 triallelic SNPs in the sequencing panel datasets at loci that had all been characterized as biallelic in the original GigaMUGA [26], along with seven biallelic sites where the alternate alleles differed.

In addition to the targeted loci, we observed 10,722 SNPs that were called in the sequencing data after applying the same filters as above (genotype quality ≥ 30, missing genotypes across samples ≤ 10%, and minimum average read depth ≥10x). Analysis of the genomic distribution of these additional SNPs indicated the vast majority (96.1%) occurred within 150bp of a target SNP (S2 Fig), suggesting that these SNPs were captured coincidentally within the span of the 150bp on-target sequencing reads. The remaining (3.80%) non-target SNPs were located considerably further away from target loci (31 kbp– 2.18 Mbp) and tended to occur in clusters, which is consistent with some degree of off-target probe hybridization as opposed to a data processing issue (e.g., mismapped reads). A substantial fraction (4,745 or 44.3%) of these SNPs had low minor allele frequencies (<0.05), and were thus filtered prior to population genetic analyses.

### Population genetic analyses

**Fig 4:**
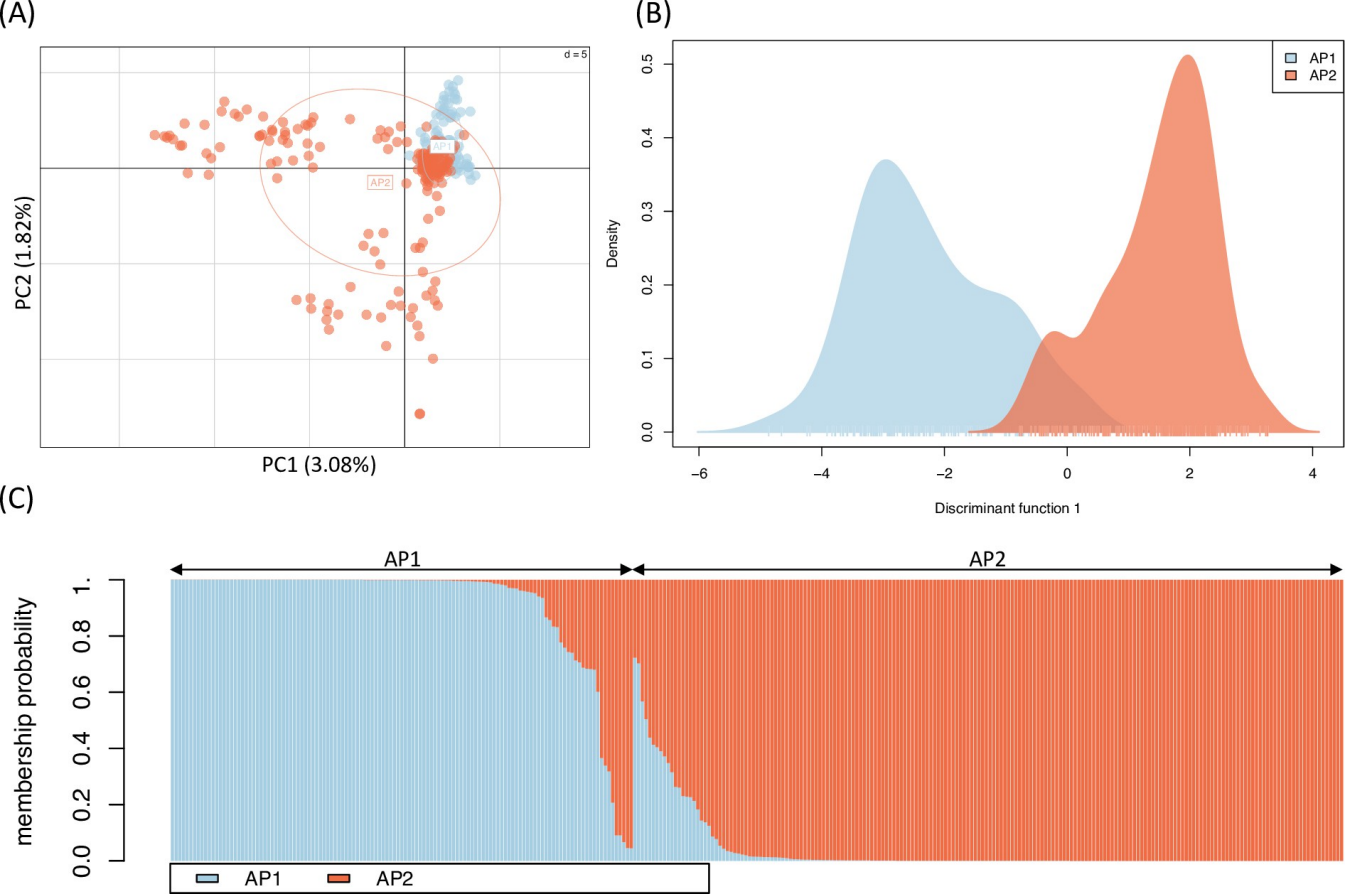
Characterization of population genetic variation based on hybridization capture sequencing dataset for house mice (N = 317) sampled from two adjoining farms in Adelaide Plains, SA (AP1 and AP2). (A) PCA, with samples plotted against first (x-axis) and second (y-axis) principal components (PCs). Percentage of total genetic variation explained by each axis provided in parentheses. (B) Density plot for individual samples (ticks on x-axis) along linear discriminant (LD) function from DAPC (25 PCs, 1 LD). (C) Posterior membership probabilities (colors) for individual mice (columns) to each population, with sampling location indicated by arrows at top of plot.

Results of PCA and DAPC for the sequencing dataset are provided in Fig 4. Inspection of the first two axes of the PCA (Fig 4A) showed a clustering of samples from AP1 compared to AP2, with some degree of overlap, though these variables accounted for only a small percentage of variation in the dataset. Based on output from the DAPC cross-validation procedure (1000 iterations per scenario), we retained 25 principal components (accounting for 28.0% of genetic variation in the dataset) which maximized the mean proportion of successful population assignments for individual samples (0.955) whilst also achieving the lowest root mean squared error (0.057). The single linear discriminant function differentiated mice from the different sites (Fig 4B), whilst plotting of membership probability for these samples (Fig 4C) shows some degree of admixture, consistent with gene flow among these neighboring populations.

**Fig 5:**
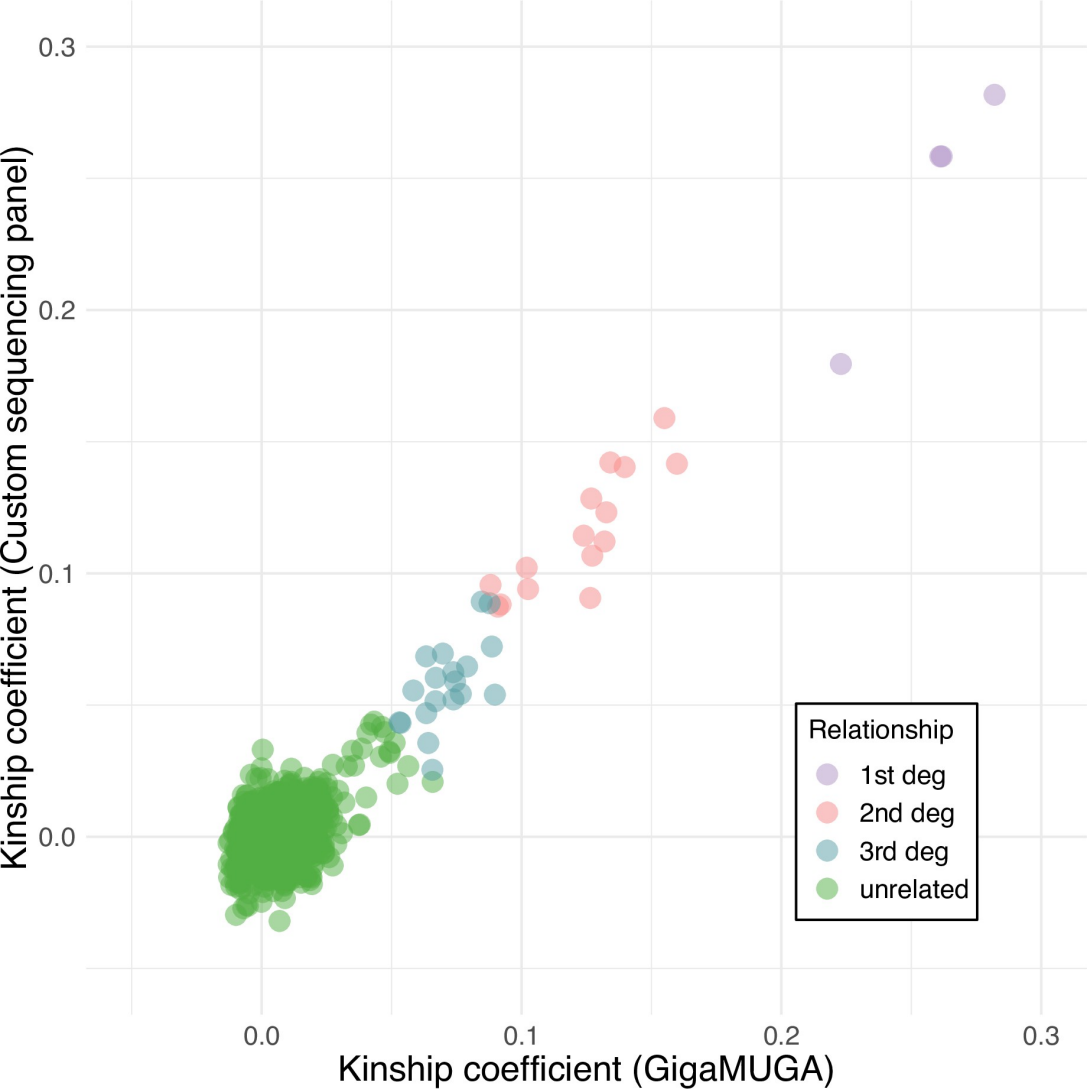
Comparison of pairwise kinship values, estimated using PC-Relate for the same samples (N = 47) using both the GigaMUGA genotyping (x-axis) and custom hybridization capture sequencing SNP datasets (y-axis). Each point represents a unique dyad, colored by the corresponding degree of kinship.

The mean pairwise kinship coefficients among the 47 individuals in the reduced sequencing dataset was relatively low (0.004, S.D. 0.024), suggesting the majority of individuals were unrelated to one another, though we detected four incidences of first degree relatives (e.g., parent/offspring, full siblings), 15 second degree relatives (e.g., half siblings), as well as 15 third degree relatives (e.g., first cousins). Estimates were strongly correlated with the values obtained from the full GigaMUGA genotypes (49,058 SNPs) for the same samples (Fig 5, Pearson’s product-moment correlation, *r =* 0.894, *t*(1079) = 65.47, *p* < 0.001).

## Discussion

Strategies for managing invasive and feral wildlife species increasingly depend on population genetic analyses to evaluate genetic structure [42], identify sources of new population outbreaks [43] and understand the evolutionary history of invasive introductions [44]. SNP datasets can be utilized at fine geographic scales to better understand fundamental aspects of breeding biology, demographic turnover, and kin structure in pest populations [45]. Yet despite rapid technological advances in sequencing technology, there is routinely a need for methods that enable quick and relatively inexpensive genotyping at the scale of hundreds to thousands of SNPs for tens to hundreds of individuals sampled across multiple populations. Efforts to monitor invasive species may also rely on periodic or opportunistic sampling that benefits from flexible processing workflows and scalability to accommodate different sample batch sizes. In this regard, genotyping arrays which target tens to hundreds of thousands of SNPs fill an important niche [28, 46], but are still relatively expensive on a per sample basis and are typically optimized for laboratory or commercial breeding lines of model species and thus the practical application to wild pest populations is often unknown. Targeted sequencing methods provide an attractive and flexible approach that can be tailored to the study populations of interest, and at least for rodent-sized genomes, can enable multiplexed sequencing of hundreds of samples at a scale suitable for benchtop sequencing platforms (e.g., Illumina MiSeq). In this study we assessed the utility of a commercially available genotyping array (GigaMUGA) and a derived custom hybridization capture sequencing panel for population genetic analyses of house mice in southeastern Australia, a key grain-producing region that is heavily impacted by aperiodic mouse plagues [20]. Mouse populations in this region have been the focus of historical and on-going research aimed at improving management practices through a better of understanding mouse population dynamics, breeding biology, and ecology [23].

Our assessment of the suitability of the GigaMUGA for studies of wild house mice yielded mixed results. While we observed overall high genotyping rates across samples, only approximately 37% of the 129,704 total autosomal markers were retained through to population genetic analyses, with nearly one third of markers in the array observed to be monomorphic in our study populations. These results closely mirror the numbers reported by other studies of wild house mice [28, 29] and undoubtedly reflect the primary design aim of the GigaMUGA for genotyping laboratory mouse lines [26]. Of particular interest for management of problem house mouse populations, we observed no evidence of alleles in the *VKORC1* coding region known to confer resistance to warfarin, despite presumed selection from the rodenticide use in Australia. This result is consistent with a previous study that reported no evidence of warfarin resistance among house mice in Western Australia [25], and might be explained by restrictions on warfarin use (e.g., permitted only around farm buildings [47]), that limits recurrent widespread exposure by mouse populations. We also note that our study queried only two SNPs, and thus the possibility of other resistance-conferring mutations in *VKORC1* or other genes cannot be ruled out from these data.

The PCA of population genetic structure with the GigaMUGA dataset revealed a hierarchical pattern of population differentiation, with genetic distances among population clusters largely mirroring the geographic distances among populations. This result is consistent with a pattern of isolation by distance (IBD), which is supported by previous studies of wild mice in agricultural settings [48] though more extensive geographic sampling will be required to fully evaluate the prevalence of IBD across the entire range.

In designing the custom hybridization capture panel presented here, we sought to include markers from the GigaMUGA that were likely to be informative across our study populations, and at a scale that would suit library preparation in a single 96-well plate format followed by sequencing on a commonly accessible benchtop sequencing instrument. Our assessment of the resulting sequencing dataset indicated a high proportion of successful target capture, with little off-target activity, and overall strong concordance with the GigaMUGA genotypes. The additional SNPs identified in the sequencing data may have some utility for applications that do not require markers to be in linkage equilibrium or the conversion of linked biallelic SNPs into multiallelic ‘microhaplotypes’ for improved power in some analyses [49]. Encouragingly, analysis of the target capture dataset using DAPC allowed us to correctly assign individual mice to respective populations with high confidence, despite evidence of gene flow among these closely located farms. Finally, in our analysis of kinship within our study populations, pairwise kinship estimated from the custom sequencing panel dataset were highly correlated with values from the GigaMUGA panel, though the former utilized an order of magnitude fewer markers than the latter. We emphasize, however, that the performance of this particular panel of SNPs may not necessarily be generalizable to house mouse populations outside of our study range, and thus the expansion to different sampling regions in the future may require further customization or tuning. Fortunately, one of the benefits of the in-solution hybridization approach utilized in this study versus other techniques (e.g., printed microarrays, amplicon sequencing) is the flexibility to add or remove probes as necessary with few anticipated impacts on panel performance.

Overall, our assessment of a commercially available SNP array suggests that, while the technology performs well and may provide a quick ‘off-the-shelf’ genotyping solution, the design focus on genotyping laboratory lines has likely created efficiency trade-offs for studies of wild mouse populations as a large majority of markers appear irrelevant to variants segregating in the wild. As an alternative, researchers interested in wild populations of other model organisms may consider the approach demonstrated here of utilizing genotyping arrays for marker discovery, which subsequently informs design of a custom targeted sequencing panel. While the taxonomic range of commercially-available genotyping arrays is relatively limited (see S3 Table), some studies have reported successful cross-species application [50, 51], with SNP conversion at scales practical for management applications. In our estimation, the efficiencies provided by the approach in this study may provide considerable cost savings over array genotyping services (e.g., est. 34–64% reduction in per sample costs, see S4 Table), especially with long-term studies where economies of scale and sequencing on high-through platforms can provide even greater efficiency.

In comparison to other genotyping-by-sequencing methods that query similar numbers of SNPs such as ddRADseq [52], per sample costs for sequencing in our study were on par with published estimates [18], whilst providing additional benefits of marker reproducibility, tolerance to sample DNA degradation, and flexibility to modify markers panels as needed. An important consideration, however, is an understanding of any biases that may be introduced as artefacts of the genotyping array design process. Notably, genotyping arrays like the GigaMUGA often suffer from strong ascertainment bias due to the marker discovery process, and care should be taken when utilizing these data (or data from derived custom sequencing panels) for some population genetic inferences [53, 54]. In the case of GigaMUGA, there is strong ascertainment bias toward SNPs occurring in the *M*. *m*. *domesticus* lineage, suggesting that analysis of wild populations that might vary in subspecies ancestry could lead to erroneous estimates of allelic diversity, and thus any population genetic inference based on such statistics should be interpreted with caution [28, 29]. We note that previous studies of subspecies composition for house mouse populations in continental Australia have indicated near exclusive *M*. *m*. *domesticus* ancestry [7, 55].

In contrast to genotyping arrays, which typically require only basic processing of tissues prior to submission to a genotyping service provider, hybridization capture protocols involve considerably more handling time for sequencing library preparation, target capture, final purification and quantification. Moreover, downstream analysis of sequencing datasets may require a greater degree of bioinformatic processing compared to analysis of array-derived genotypes. In our study, some cost efficiencies were gained by preparing libraries at 0.4X the standard reaction volume and performing sequence capture on multiplex pools of samples. At higher scales of sample throughput, some of the most labor intensive aspects might be alleviated by the use of liquid handling robots where appropriate [56], and sequencing on higher throughput sequencing platforms could allow for even greater efficiency.

In conclusion, here we have presented an evaluation of two methods for generating high-density SNP datasets in the context of applied population genetics. The most suitable method for a given study will ultimately depend on the relative constraints in terms of time, project budgets, and available expertise in molecular laboratory techniques and bioinformatics. Continual optimization of such protocols will be a key in furthering the integration of genetic insights into management of invasive and invasive pest species.

## Supporting information

S1 Table Details of custom hybridization capture sequencing panel for genotyping house mice in southeastern Australia cropping regions. (XLSX)

S2 Table Comparison of SNP genotypes generated by GigaMUGA genotyping and custom hybridization capture sequencing applied to the same subset of samples. (DOCX)

S3 Table Vertebrate species with commercially available genome-wide SNP genotyping arrays, with potential for application in wildlife/invasive species management applications. (DOCX)

S4 Table Estimated genotyping cost comparison between array genotyping and custom hybridization capture sequencing panel. (DOCX)

S1 Fig Details of DAPC for GigaMUGA genotype dataset. (DOCX)

S2 Fig Distribution of distances (in bp) between 10,722 non-targeted SNPs and the nearest targeted SNP in sequence capture dataset. (DOCX)
