# Supplementary material for: From chip to SNP: Rapid development and evaluation of a targeted capture genotyping-by-sequencing approach to support research and management of a plaguing rodent
Source: PLoS One. 2023 Aug 17;18(8):e0288701. doi: 10.1371/journal.pone.0288701 (PMC10434965; doi:10.1371/journal.pone.0288701)
Supplement: S2 Table — (DOCX) [file pone.0288701.s002.docx]

**Table S2.** Comparison of SNP genotypes generated by GigaMUGA (‘Gig’) genotyping and custom hybridization capture sequencing (‘Seq’) applied to the same subset (N=47) of Australian house mouse DNA samples. Percentages shown were calculated in relation to total number of possible genotypes at markers retained in both datasets following quality filtering (see methods). Discordant genotype counts do not include seven SNPs were the alternate alleles differed between datasets.

|  | No. SNPs | Pct.^*^ |
| --- | --- | --- |
| **total concordant genotypes** | **165,544** | **99.34%** |
|  |  |  |
| **total discordant genotypes** | **1100** | **0.66%** |
| homozygous Gig/heterozygous Seq | 604 | 0.36% |
| heterozygous Gig/homozygous Seq | 385 | 0.23% |
| homozygous for different alleles | 111 | 0.07% |
|  |  |  |
| **total missing genotypes** | **441** | **0.26%** |
| missing genotype Gig/genotyped Seq | 375 | 0.22% |
| genotyped Gig/missing genotype Seq | 46 | 0.03% |
| missing genotype in both datasets | 20 | 0.01% |

* -- Calculation details: 3,562 SNPs x 47 samples = 167,414 total possible genotypes
