# Supplementary material for: From chip to SNP: Rapid development and evaluation of a targeted capture genotyping-by-sequencing approach to support research and management of a plaguing rodent
Source: PLoS One. 2023 Aug 17;18(8):e0288701. doi: 10.1371/journal.pone.0288701 (PMC10434965; doi:10.1371/journal.pone.0288701)
Supplement: S3 Table — (DOCX) [file pone.0288701.s003.docx]

**Table S3.** Vertebrate species with commercially available genome-wide SNP genotyping arrays, with potential for application in wildlife/invasive species management applications. We note that this list is not exhaustive, and that for some species (e.g., cattle) there are numerous commercial array options available. Products listed are merely representative examples of arrays available (along with number of SNP numbers genotyped); inclusion here should not be interpreted to imply suitability for use in the methods proposed in this study.

| **Species** | **Genotyping Array (example)** | **# SNPs** | **Ref.** |
| --- | --- | --- | --- |
| Domestic chicken (*Gallus gallus*) | Axiom Genome-Wide Chicken Genotyping Array | 580,954 | [1] |
| Domestic cattle (*Bos taurus*) | Illumina BovineHD BeadChip | 777,962 | [2] |
| Domestic pig (*Sus domesticus*) | Axiom Porcine Genotyping Array | 658,692 | [3] |
| Domestic sheep (*Ovis aries*) | Illumina Ovine Infinium HD SNP BeadChip | 606,006 | [4] |
| Domestic goat (*Capra hircus*) | Axiom Caprine Genotyping v2 Array | 59,795 | [5] |
| Domestic horse (*Equus ferus caballus*) | Axiom Equine Genotyping Array | 670,805 | [6] |
| Domestic dog (*Canis familiaris*) | Axiom Canine HD Array | >710,000 | [7] |
| Atlantic salmon (*Salmo salar*) | Axiom Salmon Genotyping Array | 132,033 | [8] |
| Domestic cat (*Felis catus*) | Illumina Infinium iSelect Cat DNA Genotyping Array | 62,897 | [9] |
| Rainbow trout (*Oncorhynchus mykiss*) | Axiom Trout Genotyping Array | 57,501 | [10] |
| Water buffalo (*Bubalus bubalis* spp.) | Axiom Buffalo Genotyping Array | 89,988 | [11] |
| White-tailed deer (*Odocoileus virginianus*) | Axiom OVSNP600 Genotyping Array | 702,183 | [12] |

**References**

1. Kranis A, Gheyas AA, Boschiero C, Turner F, Yu L, Smith S, et al. Development of a high density 600K SNP genotyping array for chicken. BMC Genomics. 2013;14: 1–13. doi:10.1186/1471-2164-14-59/FIGURES/8

2. Matukumalli LK, Schroeder S, DeNise SK, Sonstegard T, Lawley CT, Georges M, et al. Analyzing LD blocks and CNV segments in cattle: novel genomic features identified using the BovineHD BeadChip. Illumina Inc: San Diego, CA. 2011.

3. Groenen M. Development of a high-density Axiom porcine genotyping array to meet research and commercial needs. Plant and Animal Genome XXIII. San Diego, CA, USA; 2015.

4. Anderson RM, McEwan J, Brauning R. Development of a high density (600K) Illumina ovine SNP chip and its use to fine map the yellow fat locus. Plant and Animal Genome XXII. San Diego, CA, USA; 2014. Available: https://pag.confex.com/pag/xxii/webprogram/Paper10725.html

5. International Goat Genome Consortium, VarGoats Consortium, Thermo Fisher Scientific. Axiom Caprine Genotyping v2 Array. Available: https://www.thermofisher.com/order/catalog/product/551344

6. Schaefer RJ, Schubert M, Bailey E, Bannasch DL, Barrey E, Bar-Gal GK, et al. Developing a 670k genotyping array to tag ~2M SNPs across 24 horse breeds. BMC Genomics. 2017;18: 565. doi:10.1186/s12864-017-3943-8

7. Canine Consortium, Thermo Fisher Inc. Axiom Canine HD Array. Available: https://www.thermofisher.com/order/catalog/product/550869

8. Houston RD, Taggart JB, Cézard T, Bekaert M, Lowe NR, Downing A, et al. Development and validation of a high density SNP genotyping array for Atlantic salmon (Salmo salar). BMC Genomics. 2014;15: 90. doi:10.1186/1471-2164-15-90

9. Gandolfi B, Alhaddad H, Abdi M, Bach LH, Creighton EK, Davis BW, et al. Applications and efficiencies of the first cat 63K DNA array. Scientific Reports 2018 8:1. 2018;8: 1–15. doi:10.1038/s41598-018-25438-0

10. Palti Y, Gao G, Liu S, Kent MP, Lien S, Miller MR, et al. The development and characterization of a 57K single nucleotide polymorphism array for rainbow trout. Mol Ecol Resour. 2015;15: 662–672. doi:10.1111/1755-0998.12337

11. Iamartino D, Nicolazzi EL, Van Tassell CP, Reecy JM, Fritz-Waters ER, Koltes JE, et al. Design and validation of a 90K SNP genotyping assay for the water buffalo (Bubalus bubalis). PLoS One. 2017;12: e0185220. doi:10.1371/JOURNAL.PONE.0185220

12. Axiom OVSNP600 Genotyping Array. Available: https://storymaps.arcgis.com/stories/9794d395588b45d7a055e86bf42d602b
