# Supplementary material for: From chip to SNP: Rapid development and evaluation of a targeted capture genotyping-by-sequencing approach to support research and management of a plaguing rodent
Source: PLoS One. 2023 Aug 17;18(8):e0288701. doi: 10.1371/journal.pone.0288701 (PMC10434965; doi:10.1371/journal.pone.0288701)
Supplement: S4 Table — (DOCX) [file pone.0288701.s004.docx]

**Table S4.** Estimated genotyping cost comparison (in AU$ as of 2022) between array genotyping (GigaMUGA) and genotyping-by-sequencing using the custom hybridization capture sequencing panel (HyCap) described in the current study (3,651 SNP targets), across a range of sample sizes. Calculations assume purified DNA as a starting point, exclude the costs of sample shipping and labor for both library preparations and running the sequencer. HyCap cost estimates include total upfront cost for synthesizing baits (at varying synthesis scales), and sequencing on an Illumina MiSeq instrument. We note that access to higher throughput sequencing platforms would offer significant per sample sequencing cost reductions for HyCap projects.

| # Samples | GigaMUGA | HyCap |
| --- | --- | --- |
| 96 | 13,515 | 8,916 |
| 384 | 54,060 | 18,782 |
| 768 | 108,120 | 42,625 |
| 960 | 135,150 | 49,202 |
