# Supplementary material for: From chip to SNP: Rapid development and evaluation of a targeted capture genotyping-by-sequencing approach to support research and management of a plaguing rodent
Source: PLoS One. 2023 Aug 17;18(8):e0288701. doi: 10.1371/journal.pone.0288701 (PMC10434965; doi:10.1371/journal.pone.0288701)
Supplement: S1 Fig — (DOCX) [file pone.0288701.s005.docx]

**Figure S1.** Details of DAPC for GigaMUGA genotype dataset. (A) Results of cross-validation procedure to determine optimal number of principal components (PCs) to retain. Plotted values indicate the proportion of samples assigned to the correct population after performing DAPC with varying numbers of PCs retained (iterated 1000 times at each value). Solid horizontal line is the median value (along with 95% CI, broken lines) of correct assignments expected by random chance. (B) Percentage of genetic variance (conserved after retaining 34 PCs) explained by the first four linear discriminants (LD1-4) identified by DAPC. (C) Assignment of individuals to populations based on DAPC results. Individual sample IDs are on the y-axis, blue crosses indicate the a priori sampling location for each individual. All individuals had a high probability (~100%) of assignment to a single population.

A)

B)d

C)d
