# Supplementary material for: From chip to SNP: Rapid development and evaluation of a targeted capture genotyping-by-sequencing approach to support research and management of a plaguing rodent
Source: PLoS One. 2023 Aug 17;18(8):e0288701. doi: 10.1371/journal.pone.0288701 (PMC10434965; doi:10.1371/journal.pone.0288701)
Supplement: S2 Fig — (DOCX) [file pone.0288701.s006.docx]

**Figure S2.** Distribution of distances (in bp) between 10,722 non-targeted SNPs and the nearest targeted SNP in sequence capture dataset.
